# Projecting the effects of long-term care policy on the labor market participation of primary informal family caregivers of elderly with disability: insights from a dynamic simulation model

**DOI:** 10.1186/s12877-016-0243-0

**Published:** 2016-03-23

**Authors:** John P. Ansah, David B. Matchar, Rahul Malhotra, Sean R. Love, Chang Liu, Young Do

**Affiliations:** Signature Program in Health Services and Systems Research, Duke-NUS Graduate Medical School, 8 College Road, Singapore, 169857 Singapore; Department of Medicine, Duke University Medical Center, Durham, North Carolina USA; Department of Health Policy and Management, Seoul National University College of Medicine, Seoul, South Korea

**Keywords:** Long-term care, Policy analysis, System dynamics, Simulation modeling, Caregivers, Labor market

## Abstract

**Background:**

Using Singapore as a case study, this paper aims to understand the effects of the current long-term care policy and various alternative policy options on the labor market participation of primary informal family caregivers of elderly with disability.

**Methods:**

A model of the long-term care system in Singapore was developed using System Dynamics methodology.

**Results:**

Under the current long-term care policy, by 2030, 6.9 percent of primary informal family caregivers (0.34 percent of the domestic labor supply) are expected to withdraw from the labor market. Alternative policy options reduce primary informal family caregiver labor market withdrawal; however, the number of workers required to scale up long-term care services is greater than the number of caregivers who can be expected to return to the labor market.

**Conclusions:**

Policymakers may face a dilemma between admitting more foreign workers to provide long-term care services and depending on primary informal family caregivers.

**Electronic supplementary material:**

The online version of this article (doi:10.1186/s12877-016-0243-0) contains supplementary material, which is available to authorized users.

## Background

The global phenomenon of population aging is expected to be accompanied by a considerable increase in the prevalence of chronic diseases and functional disabilities, particularly among older adults in developed countries. Worldwide, the population above 65 is projected to increase from 8 percent of the total population to 12 percent between 2013 and 2030 [1]. As the population of older individuals with chronic illness and disability grows, the need for assistance in activities of daily living (ADL) and instrumental ADL (IADL) from informal (i.e., unpaid) caregivers in the family will likely increase, suggesting increased demands for long-term care (LTC). Notwithstanding the benefits of informal caregiving for care recipients and its potential for reducing LTC expenditure, the impact of informal family caregiving on the health and economic well-being of caregivers also warrants attention. The trend towards a greater demand for care can potentially strain informal family caregivers who are the major source of help for dependent elderly [2]. In fact, at least 80 percent of the total hours of care provided to older adults are from informal caregivers [3]. This burden may be especially acute in Asia, where strong social and cultural norms encourage families to care for their elders, typically in their homes [4]. In Singapore, conservative welfare policies qualify a mere 0.08 percent of residents for public assistance [5], and the family is emphasized as the “first line of support” in caring for the elderly [6]. At the same time, maximal labor participation is encouraged, with citizens recognized as a key resource in the city-state [7]. This tension in policy highlights the need for policymakers and researchers to understand the impact of LTC policies on informal family caregivers, and strive for balance in labor participation and informal caregiving.

### Informal family caregiving and labor market participation

The proportion of Singaporeans ≥65 years of age is projected to more than triple from 9 percent to 29 percent between 2010 and 2050 [1]. Given Singapore’s dynamic socio-political environment and its significant demographic changes characterized by decades of sub-replacement level fertility rates and decreasing average family size [8], a remarkable increase in the magnitude of informal family caregiver burden is expected in Singapore [9]. Consistent with cultural norms, the national policy of “aging in place” in Singapore encourages families to care for their elders directly [10]. Indeed, since 1995, legislation in the form of the Maintenance of Parents Act has allowed residents above 60 who are unable to support themselves to claim monthly allowances or lump sum payment from children capable of supporting them.

The impact of caregiving on the psychological and physical wellbeing of informal family caregivers is well documented; informal caregivers were reported to have high rates of depression [11] and health care utilization [12]. In contrast, the literature on the impact of informal caregiving on labor supply remains equivocal. While several studies did not find sufficient evidence to support any significant impact of informal caregiving on labor supply decisions [13, 14], other studies have demonstrated the lower likelihood of labor market participation among informal family caregivers [15, 16]. In one of the early works that examined the impact of providing care on employment, Carmichael and Charles [17, 18] described two main mechanisms through which caring for the elderly affects the caregiver’s labor market participation – the substitution and income effects. Given a finite amount of time, the substitution effect suggests that informal caregivers are less likely to work since caregiving substitutes time and effort for paid work. On the other hand, individuals may choose to work to ensure a stable income source (income effect). The balance between the substitution and income effects determines the direction of the impact of informal caregiving on labor market participation, if any. Within this framework, one important aspect of informal family caregiving concerns the potential for informal caregivers to withdraw from the labor market. On a micro-level, this would negatively impact the household incomes of families with dependent elderly; on a macro-level, the country’s domestic labor supply is affected. Such an impact poses a potentially serious concern given the magnitude of population aging and its associated increase in informal family caregiver burden [4]. Pre-existing socio-political labor issues may be exacerbated in countries already facing a shrinking local labor supply (with a concurrent influx of foreign labor) and an increasing old-age-dependency ratio due to population aging, such as Japan, Korea, Hong Kong, and Singapore [19]. In addition, most literature on caregivers’ labor market outcomes over the past few decades come from North America and Europe [14]; there is value in exploring the Asian context, which has hitherto remained relatively unexplored, save for recent work on Japan [20] and South Korea [21]. This paper would supplement international literature with current data from Singapore, testing the relationship between caregiving and labor market participation in a new context, allowing policymakers to craft grounded measures impacting labor supply, LTC, and health services.

In Singapore, even without factoring informal caregivers’ withdrawal from the labor market, the future domestic labor supply is expected to decrease as more residents retire. To address this expected decline, Singapore sought to increase its domestic labor force productivity through improvements in technology, job-retraining programs, and encouraging females to join the workforce [22]. A landmark policy that extended the retirement age of 62 years to 65 years has also been implemented, with ongoing discussion of extending this to age 67 [23]. Despite such efforts, the projected resident labor supply is not expected to keep pace with the rising labor demand, and the share of foreigners in the labor market is still expected to increase significantly. Already, Singapore is highly dependent on foreign labor with foreigners comprising 36 percent of the country’s total labor supply [24–26]. A number of policymakers view dependence on foreign labor as an impediment to economic productivity and restructuring, raising concerns over the foreign labor influx crowding out jobs, recreational spaces, and public services [27]. Hence, ascertaining the impact of informal familial caregiving on labor force participation is of definite import to policymakers and health services providers both in Singapore, and around the region.

### Alternative long-term care options and informal caregivers’ labor market participation in Singapore

Outside informal caregiving, Singapore offers care recipients and their families three primary LTC options: (1) home- and community-based services, (2) foreign domestic workers (FDWs), and (3) nursing homes. Over time, the government aims to expand and improve the attractiveness and accessibility of home- and community-based services, enhance subsidies to increase the proportion of families that employ an FDW to take care of older adults (17 percent), and construct more nursing home spaces [28, 29].

#### Home- and community-based services

Home- and community-based services support persons with disabilities by providing center- (e.g., day rehabilitation, dementia, social and hospice daycare) and home-based services (e.g., medical and nursing services, help services, hospice care and therapy). Factors that limit the relative attractiveness of home- and community-based services include transportation difficulties to and from community centers, high service costs, inconvenient location of facilities, social connectedness and service quality [30]. To improve access to home- and community-based services, the Ministry of Health (MOH) [31] relaxed eligibility requirements to expand subsidies for families of elders with disability. Additionally, subsidies for service providers have been increased to scale up services and enhance care quality.

#### Foreign domestic workers

Almost 50 percent of Singaporean families with an ADL-limited elderly needing assistance employ an FDW [9]. This option also allows elderly to stay in familiar environments. Despite concerns about the lack of proper training in caring for frail elderly among FDWs [32], and the development of a “maid dependency syndrome” [33], the parliament has enhanced subsidies to employ FDWs for eldercare, suggesting that the future proportion of FDWs will rise [34]. Employers of FDWs can also apply for grants and concessions on levy payments for households with elderly or persons with disabilities.

#### Nursing homes

Nursing homes offer another alternative to those who have significant ADL limitations and require constant nursing care. Nursing homes provide help with custodial (e.g., bathing, getting dressed, eating) and skilled care (e.g., medical monitoring, treatments), and admit care recipients according to their level of disability and social support situation. The eligible elderly who are unable to pay are entitled to government subsidies based on a means test. The government plans to increase nursing home capacity from 9300 to 22,400 beds between 2012 and 2030 to shorten waiting lists [34, 35].

### Long-term care policies and informal caregiving: A conceptual framework

The implementation of these programs is at the center of policy debates concerning LTC in Singapore. Implementing these policies have been shown to reduce the average number of hours informal family caregivers spend per week providing care [9]. However, the ability of these LTC options to enable informal family caregivers to remain in or re-enter the labor market remains unclear. More specifically, published information on the impact of these policies on primary informal family caregiver’s labor market participation is unavailable. Estimates of the labor requirement ratios (i.e., the total number of LTC professionals required to implement the policy compared with the number of primary informal family caregivers that the policies would enable to participate in the labor market) for each of the policies may also have important implications for Singapore’s labor supply. Most LTC policies aim to increase the availability of formal (i.e., paid) care services such as nursing homes and other home- and community-based services. The range of services offered and the cost coverage for families, however, vary considerably between countries and across regions. Optimal LTC models should consider addressing the demands of fulfilling cultural expectations in caregiving within the family and minimizing the cost and burden of informal family caregiving. Nevertheless, there is a dearth of evidence on the effects of different LTC policies on outcomes relevant to informal caregivers.

In attempting to fill this knowledge gap, this study initially aims to determine the relationship between informal caregiving and labor market participation in Singapore. Establishing this relationship is fundamental in projecting the effects of LTC policies on the labor market participation of primary informal caregivers. A primary informal caregiver is defined here as the family member mainly involved in providing care or ensuring care provision to care recipients ≥60 years. Singapore provides an excellent setting for a case study given its aging population, low unemployment rate, tight labor market, and high expectations for informal family caregiver involvement in eldercare. Drawing on a system dynamics (SD) model that simulates the growth of the elderly (≥60 years) with ADL limitations needing human assistance, six LTC policy scenarios are tested, projecting their impact on the labor market participation of informal caregivers in Singapore. These scenarios include: (1) having no LTC services; (2) the current LTC policy; and alternative policy options that favor: (3) home- and community-based services; (4) FDWs; (5) nursing homes; or (6) the ‘all-in’ combination of options 3–5.

These scenarios seem peculiar to the Singapore context, and indeed the use of FDWs in caring for elderly appears quite unique. Nevertheless, other countries like Malaysia, Hong Kong, Saudi Arabia, Qatar, Kuwait, and the United Arab Emirates also see a high uptake of FDWs [36], both for domestic help and caring for dependents, including the elderly. In particular, Hong Kong faces a similar situation of an aging population. Hence, the study aims to address the following important questions for Singapore, and other countries facing an increasing elderly care burden: What will be the likely impact of the current LTC policy and other policy alternatives on primary informal family caregiver labor market participation between now and 2030? Are the policies projected to require more LTC professionals to implement than informal caregivers they enable to return to the labor market? What implications will these outcomes have for the Singapore labor market in general?

## Methods

### Data sources

Information on the prevalence of having one or more ADL limitations needing human assistance and the proportion of elderly with one to two, three to four, and five or more ADL limitations needing human assistance was obtained from the Social Isolation, Health and Lifestyles Survey (SIHLS). The SIHLS, a nationally representative survey of 4990 community-dwelling Singaporeans ≥60 years old, was conducted in 2009 by the Ministry of Community Development, Youth and Sports (MCYS; In November 2012, the MCYS was restructured to become the Ministry of Social and Family Development (MSF)). Participants of SIHLS were interviewed after written informed consent. The Singapore Survey on Informal Caregiving (SSIC), on the other hand, provided the necessary data on informal caregiving. The MCYS conducted the SSIC between 2010 and 2011, interviewing 1190 dyads of community-dwelling care recipients (i.e., Singaporeans ≥75 years receiving human assistance for at least one ADL limitation) and their primary informal caregiver (i.e., an unpaid family member or friend who is mainly involved in providing care or ensuring the provision of care to the care recipient) after written informed consent. The sampling methodology and survey details of the SIHLS [37] and the SSIC [38] are available from the literature as cited. Analysis of de-identified data from the two surveys was exempted from full review by the institutional review board of the National University of Singapore. Data on the capacity of home- and community-based services and nursing homes were obtained from publicly available sources [39, 40]. Other population and demographic data used in the model were acquired from the Singapore Department of Statistics. In the absence of available published data for model parameters, we obtained estimates from local LTC experts and policy makers. A list of selected model input parameters are presented below (Table 1).

**Table 1:** Key model input parameters

Parameter	Value			Source
Population sub-model:				
Fertility rate (used to calculate birth rate)	Total Fertility Rate Table for 2000 – 2011			Singapore Department of Statistics 2012
Mortality rate	Complete Life Tables 2006-2011 for Singapore Resident Population			Singapore Department of Statistics 2011
Care arrangement sub-model:	Number of ADL limitations	Value	Range	
Primary caregiver hours by disability caregivers without services and FDW^b^	1 – 2 ADL	31	24.8-37.2	MCYS SSIC 2010/2011
	3 – 4 ADL	36	28.8-43.2	
	≥5 ADL	42	33.6-50.4	
Primary caregiver hours by disability caregivers without FDW but with services^b^	1 – 2 ADL	25	20-30	MCYS SSIC 2010/2011
	3 – 4 ADL	48	38.4-57.6	
	≥5 ADL	37	29.6-44.4	
Proportion of families with FDW^b^	Use of FDW	Value	Range	
	With FDW	0.491	0.39-0.59	MCYS SSIC 2010/2011
	Without FDW	0.509	0.41-0.61	
Distribution of ADL limitations by age^a^	Number of ADL limitations			
	1 or 2	3 or 4	5 or 6	
60 – 64 years	0.0042	0.0069	0.0042	MCYS SIHLS 2009
65 – 69 years	0.0080	0.0109	0.0156	MCYS SIHLS 2009
70 – 74 years	0.0078	0.0230	0.0262	MCYS SIHLS 2009
75 – 79 years	0.0190	0.0427	0.0464	MCYS SIHLS 2009
80 – 84 years	0.0525	0.0892	0.0785	MCYS SIHLS 2009
≥85 years	0.1270	0.1432	0.1744	MCYS SIHLS 2009
ADL incidence rate, annual	0.00768			Model calibration
HCBS uptake rate	0.20			Estimate by LTC experts
Capacity adjustment time, years	1.5			Estimate by LTC experts
Prevalence of ≥1 ADL limitations needing human assistance in population 60+ years of age	0.0478			MCYS SIHLS 2009
Proportion of ADL-limited elderly with 1 to 2 ADL limitations needing human assistance	0.36			MCYS SIHLS 2009
Proportion of ADL-limited elderly with 3 to 4 ADL limitations needing human assistance	0.24			MCYS SIHLS 2009
Proportion of ADL-limited elderly with 5 or more ADL limitations needing human assistance	0.40			MCYS SIHLS 2009
Annual mortality rate for individuals 60 years of age and older	0.1134			Singapore Department of Statistics 2012
Nursing home beds	Year	Number of beds		
	2012	9,750		Singapore Department of Statistics 2015
	2020	14,900		MOH 1997
	2030	22,400		MOH 1997
Primary informal family caregiver labor market participation sub-model:				
Fraction of caregivers employed or employable	0.65			MCYS SSIC 2010/2011
Labor requirement estimate:				
Adjusted patient to staff ratio at nursing homes	5			Regulation and Quality Improvement Authority 2009
Patient to staff ratio for HCBS	7			Estimate by LTC experts
Patient to staff ratio for FDWs	1			Estimate by LTC experts

(*ADL* activity of daily living, *IADL* instrumental activity of daily living, *MCYS* Ministry of Community Development, Youth and Sports, *SSIC* Singapore Survey on Informal Caregiving, *HCBS* home- and community-based services, *LTC* long-term care, *SIHLS* Social Isolation, Health and Lifestyles Survey, *FDW* foreign domestic worker, *MOH* Ministry of Health)

^a^Adjusted for the number of older adults residing in nursing homes

^b^Sensitivity analyses were performed for these parameters

ADLs, as described in the SSIC, include bathing, dressing and undressing, eating, toileting, getting in and out of bed, walking, and taking care of appearance; whereas IADLs include using the telephone, getting to places out of walking distance, shopping, preparing meals, doing housework, taking medication, and handling money. The number of hours per week they helped the care recipient with one or more ADL or IADL was reported by primary informal caregivers in the SSIC. In the same questionnaire, they also reported the number of eldercare hours per week provided by other family members or FDWs. The eldercare hours provided by the primary informal *family* caregiver were calculated by subtracting the eldercare hours provided by FDWs, other family members and friends from the total reported eldercare hours. The eldercare hours per week provided by the primary informal family caregiver were then stratified by the care recipient’s number of ADL limitations.

### Regression model

The impact of informal caregiving on the labor supply is not straightforward. While the severity of a person’s disability affects the need for caregiving and may hence affect caregivers’ decisions to withdraw from or remain in the labor force, labor market opportunities that individuals are exposed to may also influence their decisions to give care full-time. For instance, those who currently have jobs face the opportunity cost of loss of income so they may be less inclined to spend more time on caregiving, whereas those who are not in the labor market may be more available to provide caregiving. To address this issue of endogeneity, we used a two-stage-least-square (TSLS) probit regression [41], an instrumental variables approach that has been used to correct for endogeneity in other studies involving cross-sectional data [16, 42]. For our study, ADL limitations categories (one to two, three to four (ADL_1_), and five or more ADL limitations (ADL_2_)) were chosen as instruments, as they are likely to be highly correlated to caregiving hours (CGH) but not directly correlated with labor force participation (LFP). In the regression models, we controlled for a vector of exogenous variables including gender (X_1_), ethnicity (Chinese or non-Chinese) (X_2_), marital status (married or not) (X_3_), having at least upper secondary education (X_4_), and having a five-room or bigger flat or private house (X_5_) using:

Stage (1)

$$ CGH\kern0.5em =\kern0.5em {\beta}_0+{\beta}_1 AD{L}_1+{\beta}_2 AD{L}_2+{\beta}_3{X}_1+{\beta}_4{X}_2+{\beta}_5{X}_3+{\beta}_6{X}_4 + {\beta}_7{X}_5+\varepsilon $$

Stage (2)

$$ P(LFP)\kern0.5em =\kern0.5em f\left({\upgamma}_0+{\upgamma}_1\widehat{CGH}+{\upgamma}_2{X}_1+{\upgamma}_3{X}_2+{\upgamma}_4{X}_3+{\upgamma}_5{X}_4+{\upgamma}_6{X}_5 + \delta >0\right) $$

where $\beta$_*i*_ and γ_*i*_ (0 ≤ *i* ≤ 7) are the regression coefficients and $\varepsilon$ denotes unobservable characteristics. The probability that the individual is in the labor market is the cumulative distribution function, *f*(.), of their caregiving hours, exogenous control variables, and a vector of unobservable characteristics, δ. $\widehat{CGH}$ is the predicted value of caregiving hours from stage 1 regression.

In our models, except for caregiving hours and age, all other covariates were coded as categorical variables. In five extreme cases where the caregivers reported spending more than 112 h per week on ADL- and IADL-related care, we recoded the caregiving hours to 112 h per week (i.e. 16 per day). Primary informal family caregivers who had never been employed were excluded in the analysis as they were unlikely to have any attachment to the labor market. Primary informal family caregivers who provided care to more than one person were also excluded to ensure reasonable comparability of all persons in the study sample. Since FDWs, when employed, were shown to provide about 70 % of caregiving hours in our study sample, we also performed the same analysis excluding care recipients who were receiving help with at least one ADL or IADL from a FDW. All the regression analyses were conducted using STATA version 13 (StataCorp, College Station, TX).

### Dynamic simulation model: long-term care model

The LTC model presented herein is a deterministic, differential, and algebraic equations-type model built using the SD methodology [43] combining local and expert evidence and insights from previous research [44, 45]. The first step in creating an LTC model involves developing a conceptual model that uses the SD iconography of stocks and flows. This conceptual model features several interacting elements, which describes essential interdependence and information feedbacks among ADL-limited elderly individuals, primary informal family caregivers, LTC services, and policies that simulate the reference modes (i.e., the behavior pattern of key variables). In this study, ten LTC experts – including healthcare planners from the MOH, patient placement agency representatives, home- and community-based service coordinators, and nursing home managers – verified the model structure, bringing evidence and expert knowledge to support strategic long-term care policy issues. Following verification, the model was parameterized using publicly available data; experts provide plausible estimates when data for a model parameter are unavailable. Finally, this model was implemented as a computer simulation using Vensim Simulation Software (Ventana Systems, Harvard, MA). The potential policy alternatives in this study were tested and the insights gained were shared with the experts.

The LTC model used in this study consists of two sub-models: the population sub-model, and the care arrangement sub-model. These sub-models provide simulations of Singapore’s resident population, trends in ADL disability among older adults, care arrangements for ADL-limited elderly requiring human assistance, and the impact of caregiving on the labor market participation of primary informal family caregivers. The model structures permit the validation of its internal structure and assumptions.

#### Population sub-model

**Fig. 1:**
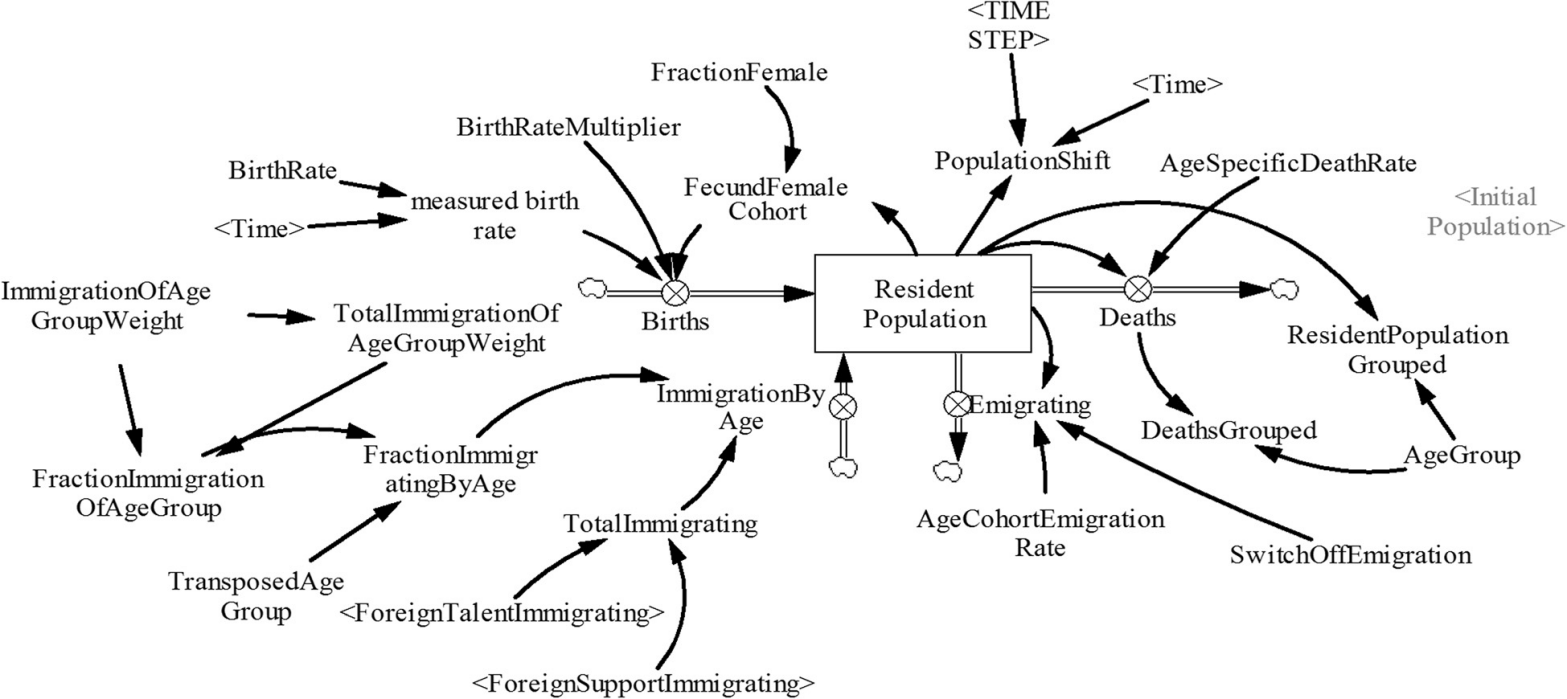
Population Model

The population sub-model (Fig. 1) disaggregates Singapore’s population into one-year age cohorts by gender. The population cohorts are affected by births (only for the first age cohort), deaths, immigration, emigration, and aging (except for the last age cohort). Births are calculated based on the fertility rate and the female population in the reproductive age group (15 – 44 years of age). Deaths are calculated using mortality rates from life tables by age. Mortality and birth rates are held constant over the simulation time. Immigration is based on available data and emigration is estimated by calibration. The aging process ensures that at the end of each year, the surviving population in each age cohort transitions to the subsequent age cohort with the exception of the final age cohort. The non-surviving population in each age cohort is removed via an outflow that reflects the respective mortality rate for that age cohort. The population sub-model is calibrated using publicly available national statistical data [46] and is described in detail elsewhere [9, 47].

#### Care arrangement sub-model

**Fig. 2:**
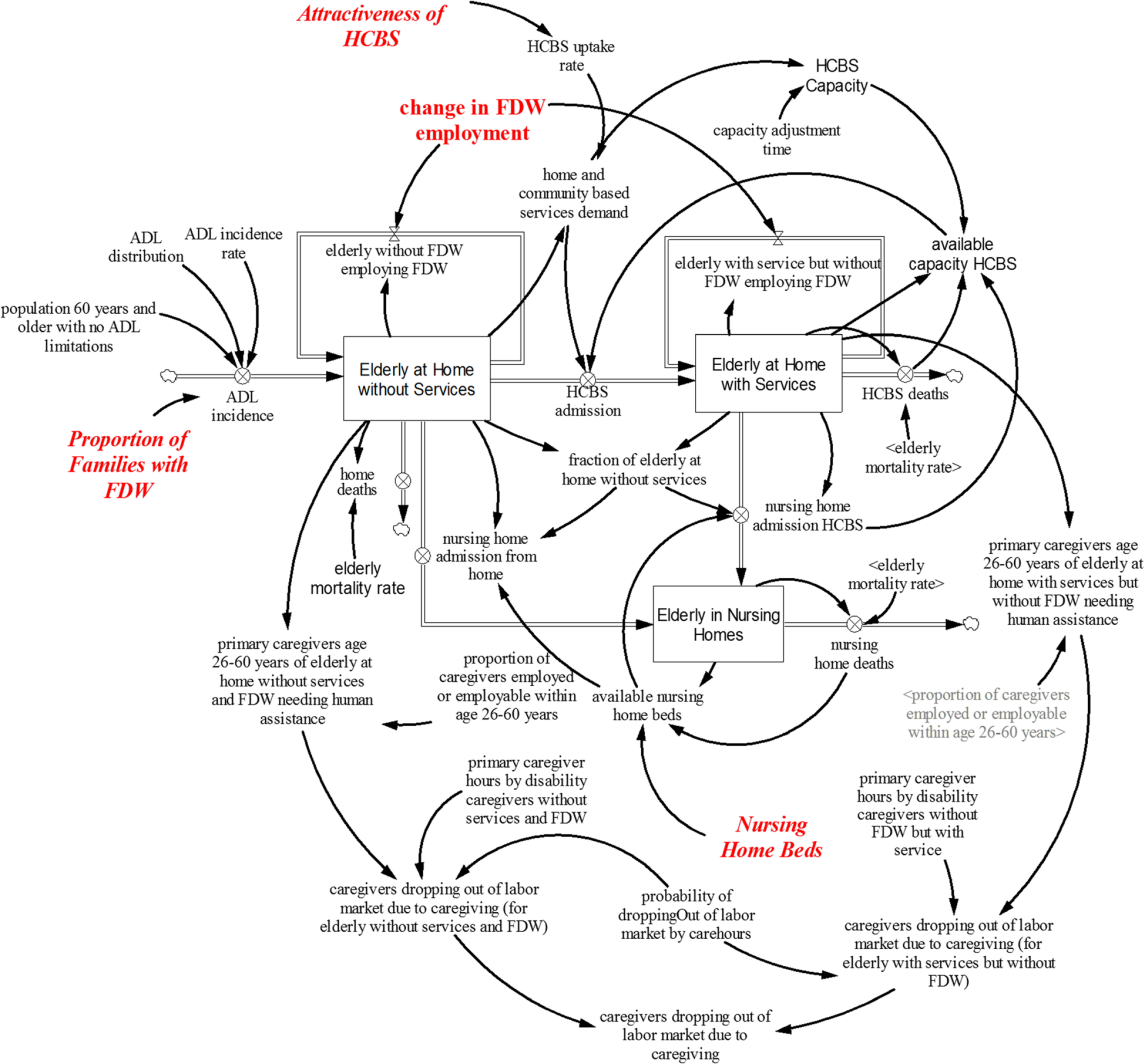
Care Arrangement Model

Inputs for the care arrangement sub-model (Fig. 2) include ADL prevalence data from the SIHLS (adjusted for the number of residents in nursing homes), data on eldercare hours for functionally limited older adults from the SSIC, and projections from the population sub-model. The care arrangement sub-model projects the elderly population with at least one ADL limitation requiring human assistance, the care arrangement of elderly with at least one ADL limitation requiring human assistance and primary caregivers likely to drop-out of the labor market due to caregiving. The projected ADL-limited elderly population requiring human assistance is disaggregated into three groups based on disability level: low (one or two ADL limitations), medium (three or four ADL limitations) and high (five or more ADL limitations).

In this sub-model, ADL-limited elderly requiring human assistance have five possible care arrangements: a nursing home; at home with home- and community-based services; at home with care provided by a FDW; at home with home- and community-based services and care provided by a FDW; and at home without any paid services. All individuals who reside at home may receive informal care (i.e., care from primary informal family caregivers and other family members and friends). The model allows individuals to move from one care arrangement to another based on preference, care needs, and the availability and capacity of services. It is assumed that available nursing home beds go to elderly individuals with the highest numbers of ADL limitations. In contrast, elderly individuals with any level of disability can choose to reside at home with or without paid services and/or make use of home and community based services. Elderly individuals residing at home are disaggregated by subscript into elderly with and without paid services (FDW).

Other assumptions include the following: nursing homes provide all eldercare hours for residents; primary care hours of caregivers using home- and community-based services is approximately half of primary caregiver hours provided by caregivers living at home without home-and community-based services. According to the SSIC, 49 percent of families with an elderly individual having at least one ADL limitation needing human assistance employ a FDW. On average, a FDW provides an estimated 70 percent of total ADL and IADL eldercare hours. Furthermore, SSIC data indicate that eldercare hours provided by families and friends were found to vary by disability. The model assumes that family members and friends (but not primary informal family caregivers) provide the elderly who have low and high levels of disability with 43 percent and 29 percent of total informal eldercare hours, respectively. To calculate the primary informal family caregiver hours by level of ADL, the number of eldercare hours provided by formal services and other family members and friends is subtracted from total eldercare hours provided to elderly individuals with ADL and IADL needs after adjusting for disability level. To calculate labor market participation of primary informal family caregivers, the number of primary informal family caregivers likely to withdraw from the labor market is estimated using the following information: the predicted probability of employment from the instrumental variable regression (see Regression model); the estimated eldercare hours provided by primary informal family caregivers; and the number of likely employable caregivers for elderly individuals with ADL limitations requiring human assistance without FDW.

Using validation, simulated projections were compared to available time series data for the total number of elderly, the total number of elderly with ADL limitations, and the total number of elderly in nursing homes in Singapore. The results (Additional file 1) proves that the projection model compares well with the time series data suggesting that on the face value, the model performs credibly.

### Long-term care scenario and policy simulations

#### No long-term care services scenario

This scenario is a counterfactual simulation in which all LTC services are assumed to be absent, and care burden is assumed to fall entirely on the family. It is used to project the number of primary informal family caregivers who would stay employed in the absence of any LTC services.

#### Current long-term care policy

This policy simulates planned or expected increases in nursing home capacity, the attractiveness of home- and community-based services, and the ability of families to employ a FDW to assist with eldercare. Under this policy, nursing home capacity increases from 9300 beds to 22,400 between 2013 and 2030. With plans to enhance subsidies to encourage the use of home- and community-based services, the uptake rate of these services is assumed to increase to 30 percent by 2030 from 20 percent in 2013. In addition, given enhanced subsidies for families employing a FDW to assist in eldercare, the proportion of families with an elderly having one or more ADL limitations needing human assistance employing a FDW to assist with eldercare is increased from 49 percent in 2013 to 60 percent by 2030.

#### Pro-home- and community-based services policy option

This policy option is identical to scenario (b) except for a significant relative increase in the attractiveness of home- and community-based services causing the uptake of home- and community-based services to increase from 20 percent in 2013 to 80 percent by 2030. This alternative policy option emphasizes home- and community-based services as a LTC option without limiting the availability of nursing homes and FDWs.

#### Pro-FDW policy option

Under this policy option, nursing home capacity and the attractiveness of home- and community-based services increase as they would in the current long-term care policy; however, the use of FDWs to assist with eldercare is emphasized. Specifically, the proportion of families employing a FDW to provide eldercare increases from 49 percent in 2013 to 80 percent by 2030.

#### Pro-nursing home policy option

Under this policy option, it is assumed that nursing home capacity begins to expand in 2013 such that all elderly individuals with five or more ADL limitations who qualify for nursing home placement are placed in a nursing home – a situation that is held constant over the simulation time. Meanwhile, the attractiveness of home- and community-based services and the proportion of families employing a FDW to assist with eldercare increase according to the current policy.

#### Combined policy option (‘all-in’)

Under the ‘all-in’ policy, the ‘pro- home- and community-based services,’ ‘pro-FDW’ and ‘pro-nursing home’ policy options are implemented simultaneously. An increase in the attractiveness of home- and community-based services causes its uptake rate to rise from 20 percent in 2013 to 80 percent by 2030. The proportion of families with a FDW assisting with eldercare increases from 49 percent to 80 percent over the simulation time; whereas the capacity of nursing homes is expanded such that from 2013 to 2030, all elderly individuals with five or more ADL limitations reside in a nursing home.

### Sensitivity analysis

Sensitivity analysis was performed on all the scenarios proposed herein to observe how a change in some of the important assumptions affect output of interest. The assumptions—proportion of families with FDW, primary caregiver hours provided by caregivers for elderly living in the community with services and FDW and for those without FDW but with services living in the community—were identified to be important assumptions. Using two-way sensitivity analysis, the values for each parameter was varied by ±20 percent, and a uniform distribution for each parameter was assumed. The model was run 1000 times, each run drew a parameter value from a uniform distribution. Next, the minimum and maximum values at 95 percent confidence level for each run were used to show the credible interval, in addition to the mean values.

## Results

From 2013 to 2030, the number of care recipient–primary informal family caregiver dyads is projected to increase from 45,600 to 100,300. Meanwhile, employed and employable primary informal family caregivers are projected to increase from 29,900 to 65,600 individuals.

The instrumental variable regression suggested that at 10, 20 and 40 primary eldercare hours per week, the percentage point reduction in labor market participation is estimated to be 11 %, 22 % and 55 % respectively for caregivers without FDW providing eldercare; however, for primary caregivers with FDW providing eldercare, no association was found between caregiving hours and dropping out of labor market. If no LTC services are available, by 2030, 27.8 percent of primary informal family caregivers (representing an estimated 1.19 percent of the total domestic labor supply) are projected to withdraw from the labor market due to caregiving (Table 2). The current LTC policy reduces the percentage of primary informal family caregivers dropping out of the labor market to 6.9 percent by 2030; under this policy, approximately 20,900 more primary informal family caregivers (or 1.08 percent of the total domestic labor supply) either remain employed or return to the labor market. The pro- home- and community-based services and pro-FDW policy options are projected to provide slight decreases (to 5.5 and 3.7 percent [0.27 and 0.18 percent of the total domestic labor supply] by 2030, respectively) relative to the current LTC policy, while the pro-nursing home and all-in policy options more reduces primary informal family caregiver withdrawal to 6.3 and 2.4 percent (0.31 and 0.12 percent of the total domestic labor supply) by 2030, respectively.

**Table 2:** Projected primary informal family caregiver labor market withdrawal, labor required and work hours lost

LTC Interventions	Caregivers in labor market		Increase in caregivers working		New paid labor to implement policy		Percentage of caregivers dropping out		Labor requirement ratio	
	2020	2030	2020	2030	2020	2030	2020	2030	2020	2030
No LTC services	23,400	37,700	—	—	—	—	28.5	27.8	—	—
	[18,800-28,000]	[29,300-45,900]					[24.4-32.9]	[23.6-32.7]		
Current policy	36,500	58,700	13,000	20,900	21,500	35,500	7.9	6.9	1.6	1.7
	[32,300-40,700]	[50,800-66,400]	[11,500-14,400]	[18,400-23,300]	[17,600-25,400]	[28,000-43,400]	[6.4-9.9]	[5.2-9.0]	[1.3-2.1]	[1.3-2.2]
Pro-HCBS	37,400	60,000	13,800	22,300	22,100	37,800	6.6	5.5	1.6	1.7
	[33,100-41,600]	[51,700-68,500]	[12,200-15,400]	[19,200-25,400]	[18,200-26,100]	[29,800-45,900]	[5.1-8.4]	[4.2-7.1]	[1.2-2.0]	[1.3-2.2]
Pro-FDW	38,700	62,000	15,200	24,100	33,600	56,600	4.5	3.7	2.2	2.3
	[34,300-43,100]	[53,400-70,300]	[13,300-17,100]	[20,700-27,800]	[28,500-38,700]	[46,200-66,900]	[3.6-5.6]	[2.9-4.6]	[1.8-2.7]	[1.9-2.9]
Pro-Nursing Home	37,400	59,200	13,900	21,500	16,400	27,200	6.5	6.3	1.2	1.3
	[33,000-41,700]	[51,000-67,400]	[12,200-15,600]	[18,600-24,400]	[13,700-19,200]	[22,100-32,600]	[5.2-8.2]	[4.9-8.1]	[0.9-1.5]	[1.0-1.6]
All-in	39,200	63,100	15,700	25,300	27,100	48,100	3.8	2.4	1.7	1.9
	[34,700-43,700]	[54,400-71,900]	[13,600-17,800]	[21,600-29,300]	[23,700-30,700]	[41,100-55,000]	[3.0-4.7]	[1.9-3.2]	[1.4-2.1]	[1.5-2.3 ]

The current LTC policy and the alternative policy options are projected to require more workers to implement than the number of primary informal family caregivers they enable to participate in the labor market (Table 2). For every primary informal family caregiver who returns to the labor market under the current LTC policy, 1.7 new paid workers are required in order to implement the policy and provide care to the care recipient (hence a labor-requirement ratio of 1.7). The pro- home- and community-based services policy option yields the same labor-requirement ratio of 1.7. In contrast, the pro-nursing home policy option is projected to yield the lowest labor-requirement ratios (1.3 whereas the pro-FDW and all-in policy options require the most new paid workers to implement and thus has the highest labor-requirement ratio (2.3 and 1.9 respectively).

## Discussion

As the number of elderly with ADL limitations needing human assistance increases, care needs and eldercare hours provided by primary informal family caregivers are projected to rise. Consequently, a proportion of primary informal family caregivers are projected to withdraw from the labor market. Compared to the size of the domestic labor supply, however, the proportion likely to withdraw due to caregiving is relatively small. Simulation experiments discussed herein show that the current LTC policy and all considered alternative policy options are projected to reduce labor market withdrawal among primary informal family caregivers. However, the current LTC policy and the considered policy options are expected to require more workers to implement than they would enable primary informal family caregivers to return to the labor market. The increase in elderly individuals with ADL limitations needing human assistance is attributable to population aging, whereas, the projected increase in primary informal family caregivers is based on the assumption that each elderly individual with care needs has a primary informal family caregiver.

The current LTC policy and the considered alternative policy options effectively reduce primary informal family caregiver labor market withdrawal. Because the number of primary informal family caregivers expected to withdraw under the current LTC policy would amount to just 0.34 percent of the total domestic labor supply, the labor market participation of primary informal family caregivers is unlikely to be a significant policy consideration from the perspective of maintaining the domestic labor supply. However, among primary informal family caregivers, because the proportion projected to withdraw from the labor market is sizeable, policymakers are apt to consider the labor market participation of primary informal family caregivers insofar as their withdrawal may have a substantial, negative impact on the income and flexibility of a significant number of families. Given that average family size in Singapore is decreasing, families are already faced with having fewer potential earners. If these projections of the proportion of primary informal family caregivers who will withdraw from the labor market in order to meet the care needs of an elder are accurate, then a considerable number of families may become dependent on the government for financial assistance—a situation that policymakers in Singapore and elsewhere generally seek to minimize.

While these findings suggest that policymaking considerations should focus more on the negative effect that primary informal family caregiver labor market withdrawal may have on family income and dependency as opposed to the domestic labor supply, analysis of the projected labor-requirement ratios under the current LTC policy and the considered alternative policy options raise other important, labor-related issues. Specifically, given the nature of the tight labor market in Singapore, policies to scale-up LTC services, including the current LTC policy, are likely to necessitate increased immigration of foreign workers and, in the case of FDWs, may lead to stresses associated with an additional person being in the household.

Compared to the current LTC policy and among the considered alternative policy options, the pro-nursing home policy option had the lowest labor-requirement ratio, meaning that this policy would require the least paid labor – domestic and foreign – to implement. This is because nursing homes are relatively more efficient, having higher care recipient to staff ratios (estimated here to be 5:1) than families with elderly with disability individually hiring FDWs (care recipient to staff ratio of 1:1). This suggests that if policymakers seek to keep the labor-requirement ratio low and to minimize foreign immigration, the optimal LTC policy would emphasize the significant expansion of nursing home capacity. However, despite the greater labor-requirement ratio, both policymakers and citizens consider the expansion of home- and community-based services to be more socially desirable given that nursing homes have capital costs, require land from a constrained supply, may engender community resistance, and are not consistent with a strong social preference for aging with family. The results presented above suggest that if policymakers in Singapore continue to make the expansion of home- and community-based services over nursing homes a priority, the efficiency of home- and community-based services as a LTC option should be maximized. This could be achieved by, for example, technological innovations, implementation designed to reduce labor requirements and improve the labor-requirement ratio of the service, and greater employer flexibility allowing caregivers to remain in the workforce.

The model presented in this paper is, to the knowledge of the authors, the first SD model that integrates population aging and the demand and supply of LTC arrangements to evaluate effects of LTC policy on labor market participation among primary informal family caregivers. SD modeling allowed for the succinct delineation of levers available to policymakers and helped demonstrate the interdependence and potential outcomes of the proposed LTC policy and various alternative policy options on primary informal family caregiver labor market participation. While the model is useful in examining the dynamics of LTC policy and the relative impact of specific options, it is not intended to be predictive. Indeed, key points of uncertainty, such as the likely future uptake of home- and community-based services are the subject of current research in Singapore.

This paper has several limitations. The predicted probability of primary informal family caregivers staying employed or returning to the labor market uses the reported, not observed, ADL and IADL related eldercare hours provided to individuals with different numbers of ADL limitations. The two-step estimation procedure employed in our analysis only uses the variability in caregiving hours induced by number of ADL limitations in the first-step estimation; therefore, the efficiency of parameter estimation could be reduced. Another related issue is that parameters from our IV estimates are applicable to those caregivers whose caregiving hours change with number of ADL limitations of the care recipient. In the model, it is also assumed that, at the population level, individual eldercare hours follow a similar trend. Studies have shown that caregivers with frequent contact or closer relationships with the disabled elderly tend to underestimate their functional ability [48, 49], leading to overestimation of their care needs. While this reporting bias can lead to skewed projections, our results reveal only a relatively small portion of primary informal caregivers dropping out of the labor market to provide eldercare. In the event that this bias is adjusted for, this number is likely to decrease, reinforcing our main finding that, in the case of Singapore, it is more likely that caregiving will not greatly impact labor force participation. In addition, other studies [15, 16] have found that there is a threshold at which eldercare hours affect the labor market participation of primary informal family caregivers. However, the data used to populate the model was unable to establish such a threshold because primary informal family caregivers were found to withdraw from labor market at all levels of eldercare hour provision. Finally, changes in the projected population will affect the number of care recipient-caregiver dyads, which may change outcomes.

## Conclusion

In sum, policymakers are faced with a difficult decision: scale-up LTC services, implying increases in the labor-requirement ratio and accepting more foreign immigration, or accept reduced primary informal family caregiver labor market participation, which may result in decreased family income and greater dependency on the state. Future work to estimate the opportunity costs of primary informal family caregivers returning to or remaining in the labor market relative to the workers hired to provide LTC would be useful in helping policymakers weigh the economic impact of the different policies considered herein. In addition, if LTC policies were to target only employed primary informal family caregivers or those who are likely to participate in the labor market, such as those who have been employed in the past, then the labor market participation of primary informal family caregivers may be greater.

### Availability of supporting data

All data from the Department of Statistics Singapore are fully available without restriction. Data collection for the SIHLS and PHASE studies were co-funded by the Singapore government therefore data release will be on an individual application basis. Interested individuals can contact Associate Professor Angelique Chan at angelique.chan@duke-nus.edu.sg.

## Additional file

Additional file 1: Model validation for selected variables. (DOCX 16 kb)

## Abbreviations

ADL: activities of daily living
FDW: foreign domestic worker
IADL: instrumental activities of daily living
LTC: long-term care
MCYS: ministry of community development, youth and sports
MOH: ministry of health
MSF: ministry of social and family development
SD: system dynamics
SIHLS: social isolation, health and lifestyles survey
SSIC: Singapore survey on informal caregiving
TSLS: two-stage-least-square
