# Supplementary material for: Projecting the effects of long-term care policy on the labor market participation of primary informal family caregivers of elderly with disability: insights from a dynamic simulation model
Source: BMC Geriatr. 2016 Mar 23;16:69. doi: 10.1186/s12877-016-0243-0 (PMC4806512; doi:10.1186/s12877-016-0243-0)
Supplement: Additional file 1: — Model validation for selected variables. (DOCX 16 kb) [file 12877_2016_243_MOESM1_ESM.docx]

**Additional Figure 1. Behavior validation for total number of elderly in Singapore.**

**Additional Figure 2. Behavior validation for total number of elderly with ADL limitations in Singapore.**

**Additional Figure 3. Behavior validation for total number of elderly nursing homes in Singapore.**
